# Thermal, Mechanical and Chemical Analysis of Poly(vinyl alcohol) Multifilament and Braided Yarns

**DOI:** 10.3390/polym13213644

**Published:** 2021-10-22

**Authors:** Tania F. Freire, Tiago Quinaz, Aureliano Fertuzinhos, Nguyễn T. Quyền, Marcelo F. S. M. de Moura, Marcos Martins, Andrea Zille, Nuno Dourado

**Affiliations:** 1 CMEMS-UMinho, Departamento de Engenharia Mecânica, Campus de Azurém, Universidade do Minho, 4804-533 Guimarães, Portugal; taniaferreirafreire14@gmail.com (T.F.F.); quinaz.tiago@gmail.com (T.Q.); afertuzinhos@dem.uminho.pt (A.F.); mmartins@dei.uminho.pt (M.M.); 2 2C2T-Centro de Ciência e Tecnologia Têxtil, Campus de Azurém, Universidade do Minho, 4804-533 Guimarães, Portugal; quyen@2c2t.uminho.pt (N.T.Q.); azille@2c2t.uminho.pt (A.Z.); 3 Departamento de Engenharia Mecânica, Faculdade de Engenharia da Universidade do Porto, 4200-464 Porto, Portugal; mfmoura@fe.up.pt

**Keywords:** polyvinyl alcohol, braided yarns, dynamical mechanical analysis, viscoelastic properties, creep and relaxation, textile

## Abstract

Poly(vinyl alcohol) (PVA) in multifilament and braided yarns (BY) forms presents great potential for the design of numerous applications. However, such solutions fail to accomplish their requirements if the chemical and thermomechanical behaviour is not sufficiently known. Hence, a comprehensive characterisation of PVA multifilament and three BY architectures (6, 8, and 10 yarns) was performed involving the application of several techniques to evaluate the morphological, chemical, thermal, and mechanical features of those structures. Scanning electron microscopy (SEM) was used to reveal structural and morphological information. Differential thermal analysis (DTA) pointed out the glass transition temperature of PVA at 76 °C and the corresponding crystalline melting point at 210 °C. PVA BY exhibited higher tensile strength under monotonic quasi-static loading in comparison to their multifilament forms. Creep tests demonstrated that 6BY structures present the most deformable behaviour, while 8BY structures are the least deformable. Relaxation tests showed that 8BY architecture presents a more expressive variation of tensile stress, while 10BY offered the least. Dynamic mechanical analysis (DMA) revealed storage and loss moduli curves with similar transition peaks for the tested structures, except for the 10BY. Storage modulus is always four to six times higher than the loss modulus.

## 1. Introduction

The most commonly used techniques to repair bone injuries involve the use of mechanical fixation systems that employ wires, screws, grids, or metal plates (i.e., osteosynthesis plates) [1]. Osteosynthesis plates are highly compliant with the anatomy of each patient and are employed to ensure mechanical stabilisation and fracture alignment, which enhances bone tissue regeneration [2,3,4]. However, the fixation of osteosynthesis metal plates fastened with bicortical screws can lead to irritation and necrosis of the skin of the patient [5,6]. Other failure occurrences associated with those fixation systems are due to bone fatigue in threaded joints or loosening of the locking interface, proving to be inefficient in healing some types of fractures due to deficient interfragmentary motion [7]. Thus, it is important to study and develop an alternative fixation system apt to respond adequately to temporary bone injuries while implying minimally invasive surgical procedures compared with currently used fixation systems. Numerous solutions could be developed using hydrophilic water-soluble synthetic polymer polyvinyl alcohol (PVA) in a fibrous form if its mechanical behaviour (both monotonic and viscoelastic) was sufficiently known. Those solutions could be customised with a proper fibrous architecture of PVA according to bone fracture configuration, fragment size, and location. They would also benefit from intrinsic PVA features such as biocompatibility, biodegradability, and odourlessness, while preserving the required mechanical performance of the fastening system to allow the regeneration of bone tissue throughout the clinical recovery period.

PVA is a water-soluble and biocompatible synthetic homopolymer [8,9,10,11], which presents a low friction coefficient, low interfacial tension, high fluid permeability, high elasticity, and hydrophilicity [10], and it is a non-toxic polymer [12]. Due to its good chemical resistance, thermal and mechanical characteristics (exhibits a non-linear mechanical behaviour under tensile and compressive loading [13]), PVA is widely used in several biomedical applications, namely in glucose sensors, immunosuppressive membranes, artificial cartilages, contact lenses, drug delivery systems [14], as well as for the repair of osteochondral defects [15] and implants [16]. It has been proven that the higher the molecular weight of PVA, the greater its crystallinity and, consequently, the greater the thermal stability and better mechanical tensile strength. Furthermore, it is known that the lower the percentage of hydrolysis, the greater the flexibility and water solubility at low temperatures, and the lower the mechanical tensile strength of PVA [11].

PVA can be used in different ways, the most common being in the form of membrane [14,17] and hydrogel [8,9,16] at a low cost [10,12]. The physical and chemical properties of PVA depend on synthetic conditions and the degree of hydrolysis of the polymer itself.

Braided textiles have achieved fast-growing importance in the reinforcement of composite materials and biomedical applications nowadays [18,19]. Braids constitute textile structures that present several advantages over other biotextile structures [20]. They are semipermeable tubular structures [20] that present natural high flexibility [18,19,20] and high tensile strength in the longitudinal direction [19]. Braided textiles are also dimensionally stable, present moderate stiffness [18] and porosity [18,21], and exhibit a typical high load capacity to weight ratio [18]. An aspect of utmost importance regards the possibility to apply braided structures in in vivo therapeutics (i.e., biomedical applications) through a catheter or trocar without causing significant challenges or complications [20]. A distinctive parameter of a braided structure is its braid angle, whose amplitude lies in the interval of 20–80°, while typical patterns are (a) the regular braid (2/2), which is the most common; (b) the diamond braid (1/1) intersection repeat; (c) and the Hercules braid (3/3) intersection repeat. Moreover, there is also the distinction between biaxial and triaxial braids. The difference is the presence of axial yarns in a straight position (0°) in the direction of production (longitudinal direction) inside the triaxial braided textile. The biaxial braided textile does not have axial yarns [22]. Axial yarns or 0° reinforcements are inlay yarns that remain inactive in the production phase and are surrounded by the crimped braided yarns [18,22]. Previous research has used braided structures in combination with porous polymeric materials, proving that those composite structures are in effect stable [23].

PVA scaffolds have been studied by Teixeira et al. [11], offering high tensile strength and elongation at break, which is particularly suitable for the repair of bone fractures. Jain et al. [24] analysed the effect of stress and temperature on creep and recovery behaviour of both pristine and crosslinked PVA. Creep test results put into evidence that crosslinked composites show greater creep stiffness compared to pure PVA at different stress levels. At higher temperature levels, creep strain increases extensively for PVA. It has been proved that the non-recovered creep deformation of pristine PVA is higher compared to the crosslinked compound, which shows a higher viscous nature of pristine PVA [25]. In another study, Zhao et al. [26] analysed the effect of different types of PVA fibres on creep behaviour (e.g., steel, PVA, polypropylene, and basalt fibres). Those authors concluded that the elastic modulus of fibres is the significant factor that influences creep, and fibres with a greater modulus of elasticity can restrict creep behaviour. The rheological behaviour of PVA was investigated in an aqueous solution and in the hydrogel state. Bercea et al. [27] found that in PVA solutions, shear-induced aggregation at 37 °C and PVA hydrogels present a high elasticity and stability due to the strong polymer–polymer interactions established between the polymer chains. According to those authors, PVA/polyurethane composite hydrogel presents high elasticity up to shear stress of 3 MPa, being followed by a rapid recovery of the hydrogel structure after showing successive levels of deformation. Thus, this composite containing PVA exhibits self-healing ability, demonstrating its ability for tissue engineering applications [16].

The former works do not cover the mechanical behaviour of PVA-braided structures neither for the quasi-static nor the viscoelastic response. Taking into account this gap in the literature, monotonic quasi-static tensile tests were performed in different PVA structures to evaluate the material strength, the Young modulus, ultimate stress *σ^u^*, and ultimate strain *ε^u^*. Viscoelastic characterisation was also executed through creep and relaxation tests and dynamic mechanical analysis (DMA).

## 2. Materials and Methods

### 2.1. Materials

PVA six-filament yarn (Mintval^®^, Kuraray, Tokio, Japan) was obtained by the melt spinning process of Exceval^®^ polymer from Kuraray (Tokio, Japan). All the other reagents used for characterisation were analytical grade purchased from Sigma–Aldrich^®^, St. Louis, MO, USA and used without further purification.

### 2.2. Preparation of the Braided Yarns

**Figure 1:**
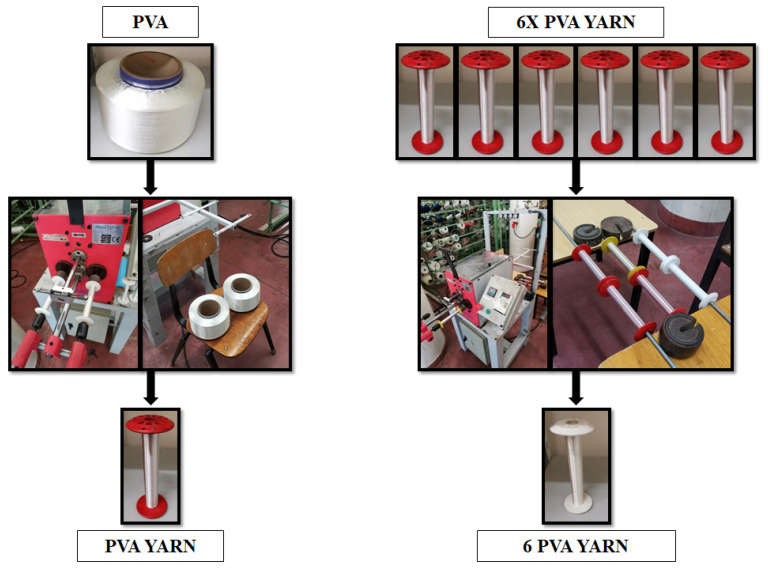
Production process of 6 PVA yarn.

PVA yarn constituted by six filaments (i.e., PVA multifilament yarn) is the building block material of this work. PVA multifilament yarns were transformed into PVA multifilament yarn bobbins. Six of them were joined to form a six-yarn bobbin (i.e., 6 PVA yarn with 36 filaments). Both stages were executed in a TRENZ-EXPORT^®^ (Barcelona, Spain) winding machine, model PR/810, serial number 00/2311 (Figure 1).

**Figure 2:**
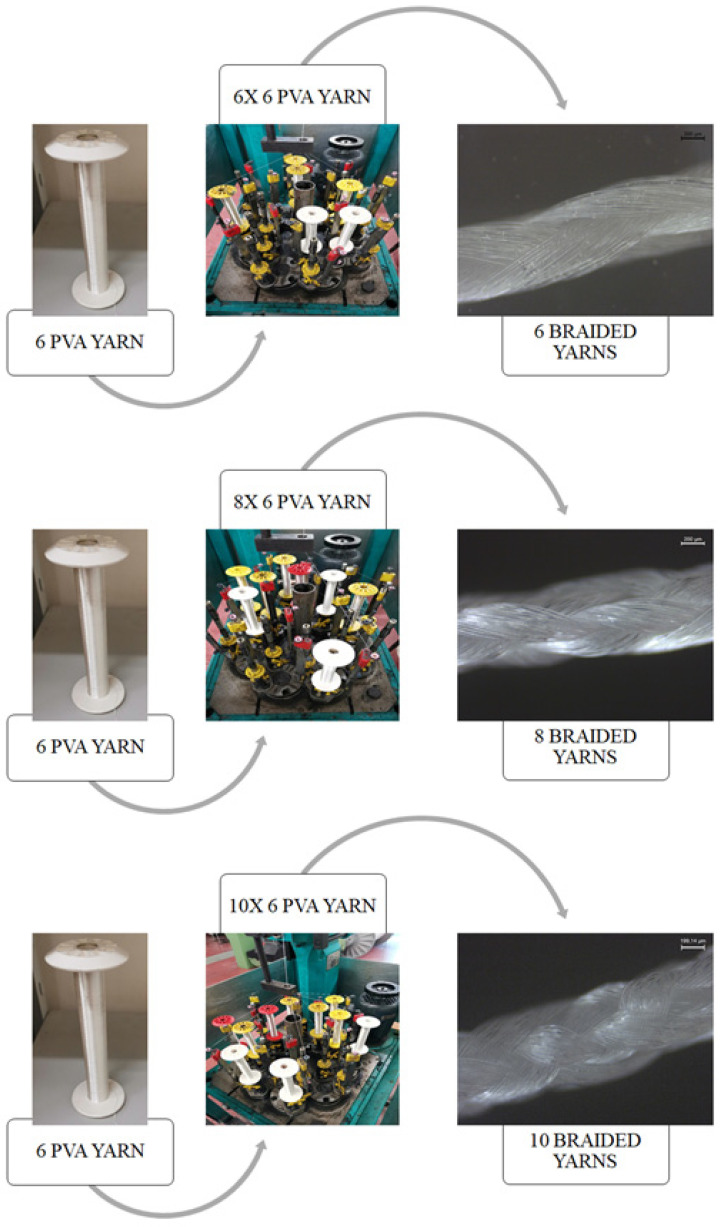
Production process of PVA 6, 8, and 10 braided yarns and the respective microscopic images.

Three types of braided yarns (6, 8, and 10) were formed from 6 PVA yarn (Figure 1). This process was executed in a TRENZ-EXPORT^®^ (Barcelona, Spain) vertical braiding machine, model 16/100, serial number 00/2295. Figure 2 shows the braiding process.

### 2.3. Scanning Electron Microscopy (SEM) Observation

The morphology of each PVA architecture was analysed by scanning electron microscopy (SEM), an ultra-high resolution field emission gun scanning electron microscopy (FEG-SEM), NOVA 200 Nano SEM, FEI Company (Hillsboro, OR, USA). Secondary electron images were performed at an acceleration voltage of 10 kV. Samples were covered with a thin film (25 nm) of Au-Pd (80–20 weight %), using a high-resolution sputter coater, 208HR Cressington Company (Watford, UK), coupled to an MTM-20 Cressington High-Resolution Thickness Controller. 

The braiding angle of each fibrous architecture, a fundamental physical property, was accurately measured from images obtained by SEM, using the ImageJ software.

### 2.4. Thermogravimetric Analysis (TGA) and Differential Thermal Analysis (DTA)

Thermal analyses were carried out in an STA 7200 Hitachi^®^ (Fukuoka, Japan) in which TGA and DTA are shown simultaneously. PVA yarn (1Y) was submitted to a single heating step within the range of 25–500 °C under nitrogen atmosphere (200 mL min^−1^) at 3 °C∙min^−1^ using an aluminium pan. The initial mass was measured prior to testing. TGA data were plotted as weight loss (WL) (in percentage) versus temperature. The derivative thermogravimetric (DTG) analysis was performed to identify the thermal transformation events (namely the maximum peaks). DTA data were plotted with ∆*T* versus temperature.

### 2.5. Fourier Transform Infrared (FTIR) 

FTIR spectra of the PVA yarn (1Y) was collected using a Shimadzu^®^ spectrometer, model FTIR-8400S, IRAffinity-1 (Kyoto, Japan), coupled with an attenuated total reflectance (ATR) accessory, the PIKE MIRacleTM single reflection with a diamond/ZnSe crystal (PIKE Technologies, Madison, WI, USA). Spectra were obtained in the range of 4000–700 cm^−1^ from 30 scans at a resolution of 4 cm^−1^. All measurements were performed in triplicate. 

### 2.6. Monotonic Tensile Tests

Six PVA yarn architectures were subjected to uniaxial tensile testing to rupture: one yarn (1Y), six parallel yarns (6PY), six PVA yarn (6Y), six, eight, and ten braided yarns (6BY, 8BY, and 10BY, respectively) to evaluate quasi-static properties. A total of 28 specimens were tested per PVA fibrous architecture.

**Figure 3:**
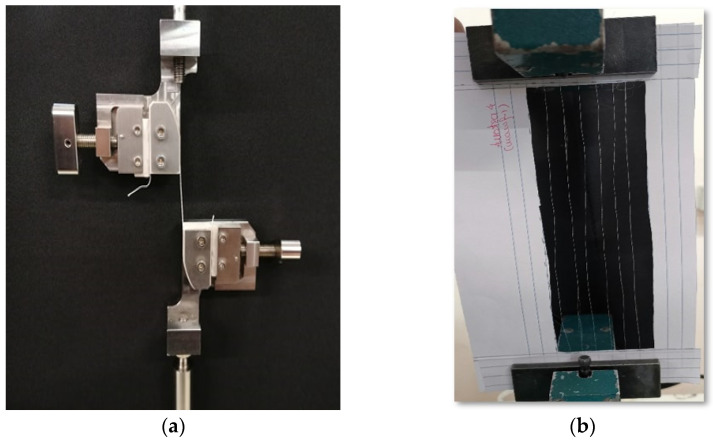
Gripping systems used for monotonic quasi-static tensile tests: (**a**) for most configurations; and (**b**) for 6PY configuration.

Tensile tests were performed in a testing machine H100KS (Tinius Olsen/Housefield, Salfords, UK) with a 250 N load cell at a 100 mm/min crosshead velocity. Load–displacement curves were obtained to evaluate the initial stiffness (*K*_0_), ultimate load (*P*_u_), and displacement at rupture (*δ*_u_). Before testing, a low tare load (0.1 N) was applied to establish a consistent zero position. Then, the adjusted distance between grips was used as the initial length. The gauge length was set to 100 mm, except for the 6PY, with 170 mm (Figure 3).

### 2.7. Creep Tensile Tests

Three PVA braided yarn architectures—6BY, 8BY, and 10BY—were subjected to creep tensile tests to evaluate their viscoelastic properties. A step function with magnitude *σ*^0^ was defined with a creep time of 7200 s, with an acquisition frequency set to 5 Hz (maximum variation during the creep analysis of ±1%). The stress magnitude was set equal to 16% of the tensile strength *σ*^u^ (true value) previously evaluated in uniaxial tensile tests for each braid geometry. A total of 10 specimens were tested per braided architecture (6BY, 8BY, and 10BY).

### 2.8. Relaxation Tensile Tests

Tensile relaxation tests were performed in the braided configurations (i.e., 6BY, 8BY, and 10BY) to evaluate viscoelastic properties. A step function with magnitude *ε*_0_ was fixed for a relaxation time of 2700 s and an acquisition frequency of 5 Hz. The strain magnitude was set equal to the strain obtained when stress attained 16% of the tensile strength *σ*^u^ (true value). A total of 10 specimens were also tested per braided architecture (6BY, 8BY, and 10BY).

### 2.9. DMA

Dynamic mechanical analyses were performed in a 7100 DMA from Hitachi^®^ (Fukuoka, Japan) in the programmed tension (tensile) method. These analyses were carried out in an atmosphere of nitrogen (200 mL min^−1^) to ensure an inert environment. The presented values for tension moduli were recorded over a range of frequencies from 0.1 to 2 Hz in synthetic oscillation. Temperature dependence of storage and loss moduli (*E*′ and *E*″, respectively), and corresponding loss tangent, tan*δ*, was measured in the range of 25 to 160 °C at 3 °C min^−1^ (i.e., the heating rate).

## 3. Results and Discussion

### 3.1. Scanning Electron Microscopy (SEM) Observation

**Figure 4:**
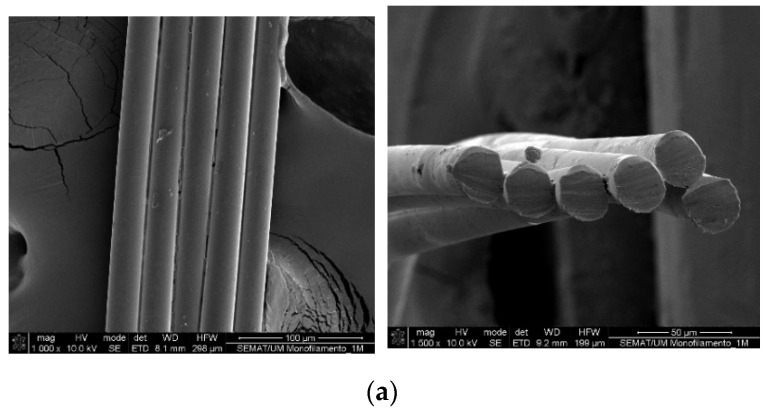

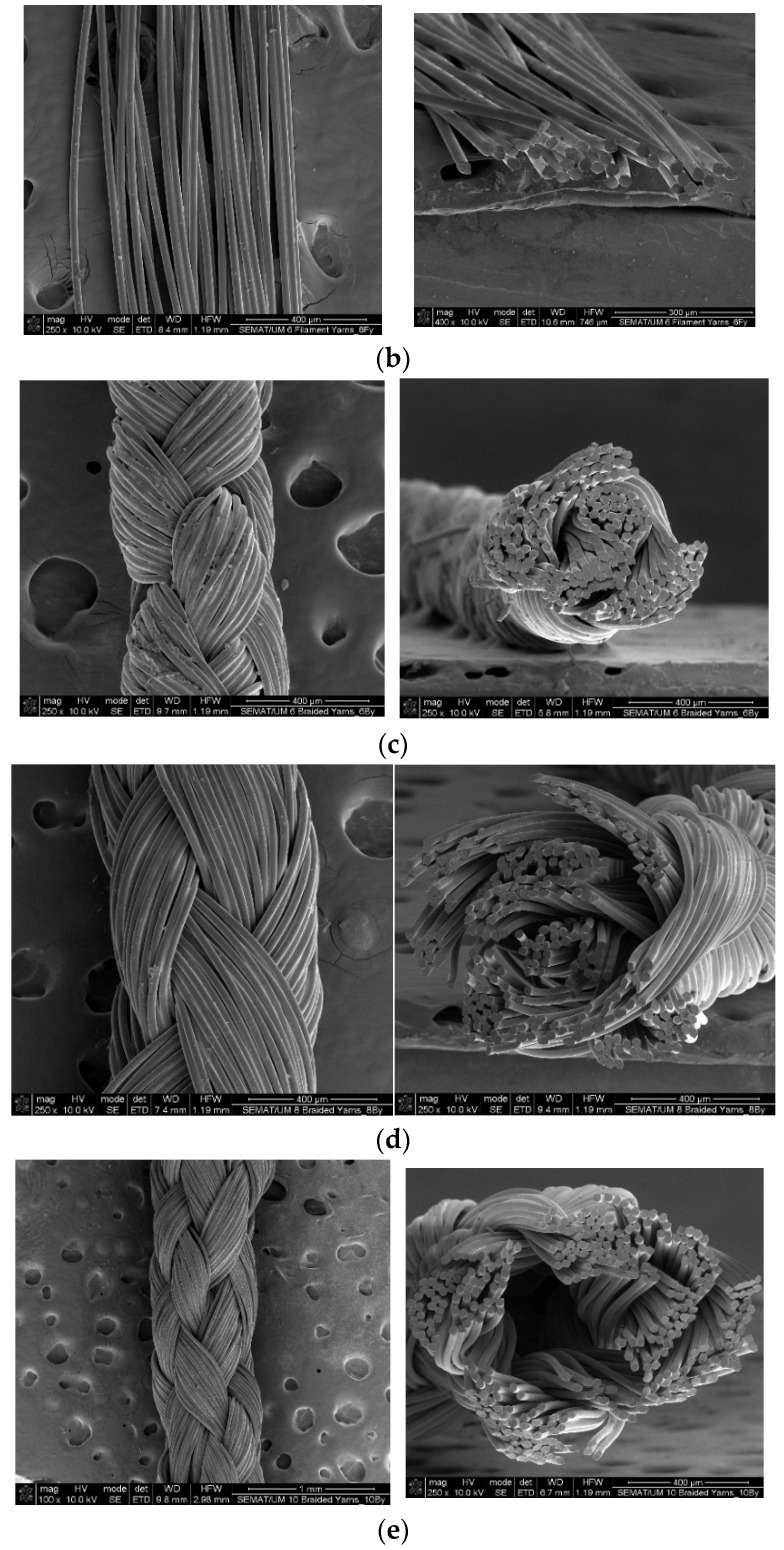
SEM images of the longitudinal surface of different PVA architectures with corresponding transverse section: (**a**) 1Y, (**b**) 6Y, (**c**) 6BY, (**d**) 8BY, and (**e**) 10BY.

Figure 4 shows SEM images of the analysed PVA configurations. It is evident that the 1 PVA yarn (1Y in Figure 4a) contains six filaments. The number of filaments will be referred to throughout this work (Table 1). Figure 4c–e demonstrate that 6BY, 8BY, and 10BY are biaxial braided structures and exhibit the same diamond pattern (1/1).

The braiding angles of the three braided architectures studied in this work were measured from the SEM images and using the ImageJ software, as shown in Table 1.

### 3.2. TGA

**Figure 5:**
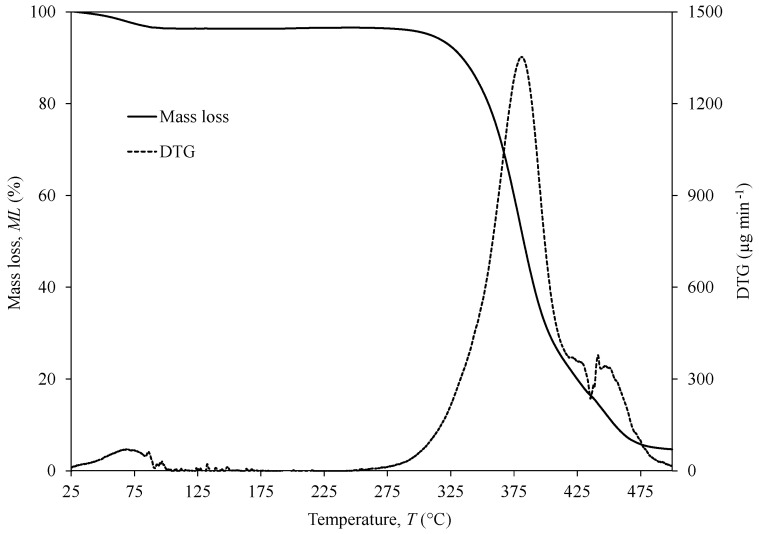
TGA (continuous line) and DTG (dashed line) of 1Y PVA from 25 to 500 °C.

TGA results for PVA 1Y configuration revealed a first small weight loss (WL) up to 100 °C (Figure 5), which is associated with water evaporation due to the hydrophilic nature of PVA. The degradation of the PVA 1Y occurs in two steps with a DTG temperature peak at 385 °C (dashed line in Figure 5) corresponding to 90% of WL and a small DTG peak at 450 °C (approximately 20% of WL). At the end of the experiment (500 °C), only a tiny, carbonised residue content of 5% remains. The main WL at 385 °C corresponds to the PVA main chain dehydration reaction, while the small peak at 450 °C is related to the polyene residue degradation [28]. The relatively high degradation temperature compared to pure PVA suggests higher thermal stability of the PVA fibres due to the higher orientation and crystallinity that fibre configuration offers or due to a possibly crosslinking reaction during the preparation of the commercial fibres [29].

### 3.3. DTA

**Figure 6:**
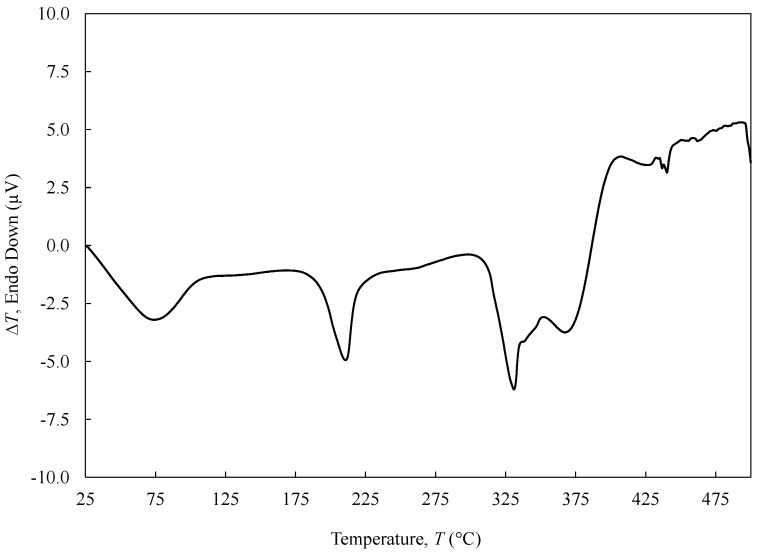
DTA of 1Y PVA from 25 to 500 °C.

Four main endothermic peaks were observed in the DTA of PVA fibre (Figure 6). The DTA thermogram exhibits a first endotherm broad peak centred at around 76 °C corresponding to the glass transition temperature (*T*_g_) of PVA in accordance with the DMA data [30]. No other peaks are observed below the *T*_g_ temperature. Only DMA analysis is sufficiently sensitive to depict beta transitions (see Section 3.8). A second endotherm peak corresponding to the crystalline melting point (*T*_m_) of PVA was observed at 210 °C. The degree of crystallinity of PVA 1Y was estimated to be 30% and was determined as the ratio between the calculated heat of fusion (∆*H*_f_; 47 J g^−1^) and the thermodynamic enthalpy of melting of a 100% crystalline PVA (∆*H*_0_; 150 J g^−1^) [31]. The third and fourth peaks at 330 and 370 °C are related to the PVA degradation of the C–C main chain, the elimination of hydroxyl groups as water, and the formation of polyene macromolecules [32]. However, the presence of two expressive peaks at such close temperatures could indicate the presence of crosslinked side-chain degradation [33,34].

### 3.4. FTIR

**Figure 7:**
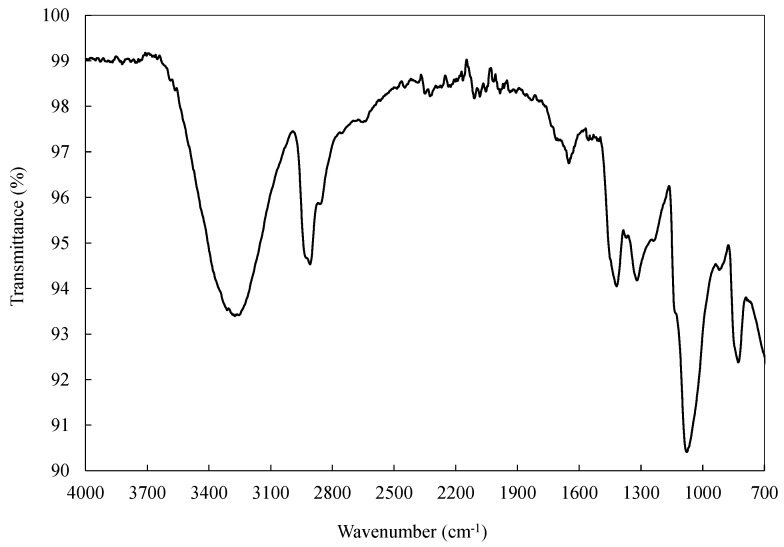
ATR-FTIR spectrum of PVA 1Y.

FTIR analysis was performed to provide information about the PVA 1Y structure and the presence of specific chemical groups in the material (Figure 7). The PVA fibres showed the characteristic absorption peaks of pure PVA at 3380 cm^−1^ attributed to the stretching ν(O–H), 2910 and 2880 cm^−1^ are related to the stretching of ν(C–H2) and ν(C–H), respectively, 1736 cm^−1^ is attributed to the stretching of ν(C=O), 1420 cm^−1^ is due to the bending of δ(CH–O–H), 1320 cm^−1^ is attributed to the wagging π(C–H), 1080 cm^−1^ is related to the stretching of ν(C–O), and 830 cm^−1^ is due to the stretching of ν(C–C) [12,28]. The expressive absorption band at 1650 cm^−1^ could be attributed to water adsorbed in the PVA fibre. Nonetheless, it can also be related to a partial interaction of the OH groups of PVA with a crosslinker [12]. Moreover, the appearance of the two shoulders at 1720 and 1260 cm^−1^ attributed to the stretching of ν(C=O) and ν(C–O–C), respectively, could be attributed to a low degree of hydrolysis or to a possible crosslinking of the PVA [35,36]. The presence of a low degree of hydrolysis can explain the beta relaxation (*T*_β_) peak observed in the DMA analysis (see Section 3.8) due to the acetate groups still present in the PVA structure [37].

### 3.5. Monotonic Tests

**Figure 8:**
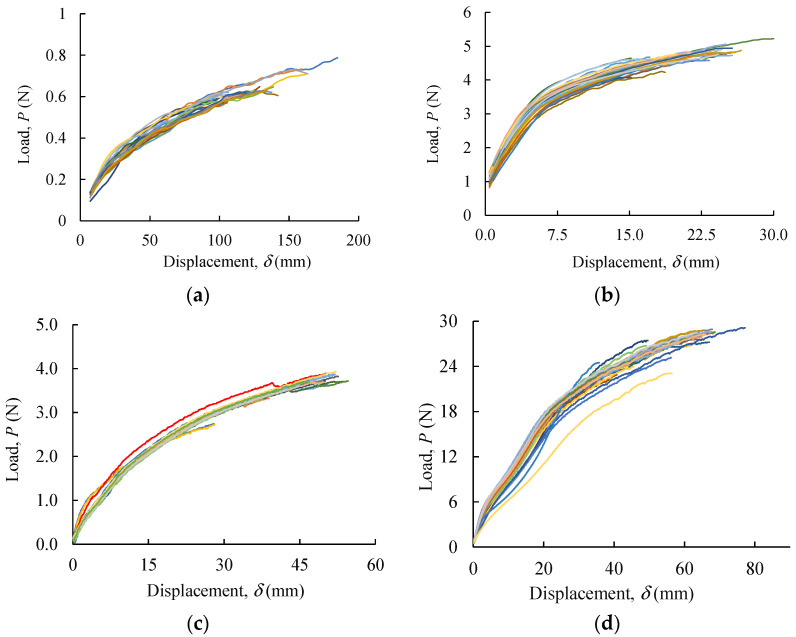

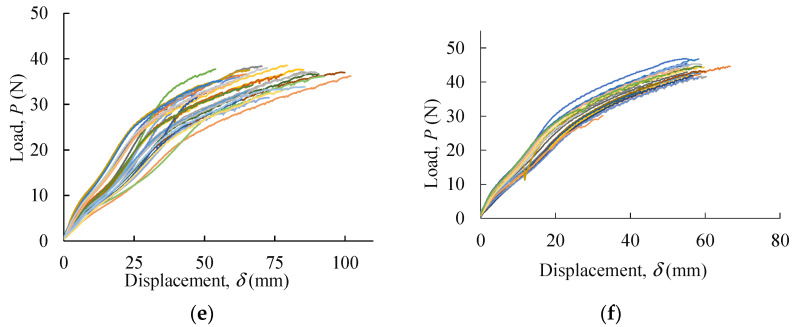
Load–displacement curves of PVA structures: (**a**) 1Y; (**b**) 6PY; (**c**) 6Y; (**d**) 6BY; (**e**) 8BY; (**f**) 10BY.

Uniaxial tensile tests were executed to evaluate the elastic stiffness (*K*_0_), ultimate load (*P*_u_), and displacement at rupture (*δ*_u_) of the studied PVA architectures (Figure 8a–f). It can be noticed that the results are very consistent with each other, which is revealed by the minor scatter of the *P*–*δ* curves. Mean values were calculated from the above-referred parameters and presented in Table 1.

**Figure 9:**
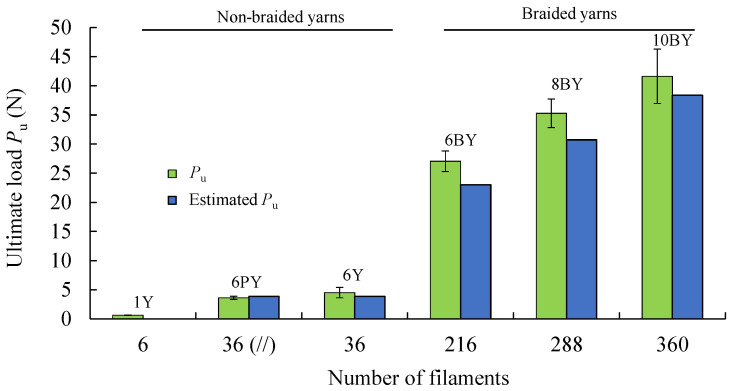
Comparison of mean values (from 28 results) of ultimate load (*P*_u_) as a function of the number of PVA filaments used in the analysed structures.

**Figure 10:**
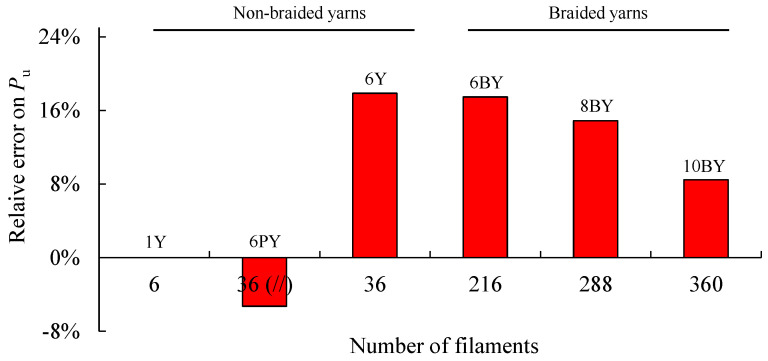
Quantitative analysis of relative resistances error as a function of the number of PVA filaments.

Figure 9 shows the evolution of *P*_u_ with the number of filaments (6 to 360, in Table 1), showing a clear trend. The first three configurations in this figure correspond to non-braided yarns, while the last three concern braided structures. To assess the influence of friction, a test was carried out in the 6PY configuration, with the same number of filaments as the 6Y, but in this case, they were spaced apart and parallel to each other, as shown in Figure 3b. The obtained results have shown no significant differences between 6PY and 6Y configurations (i.e., parallel, and wound yarns, respectively), although these are not neglectable. This figure also allows observing the difference between the attained experimental value of *P*_u_ and the value estimated considering the resistance offered by a single filament of PVA multiplied by the number of filaments (Table 1). Hence, the relative resistances error is shown in Figure 10, which renders it possible to conclude that this error decreases with the number of filaments.

**Figure 11:**
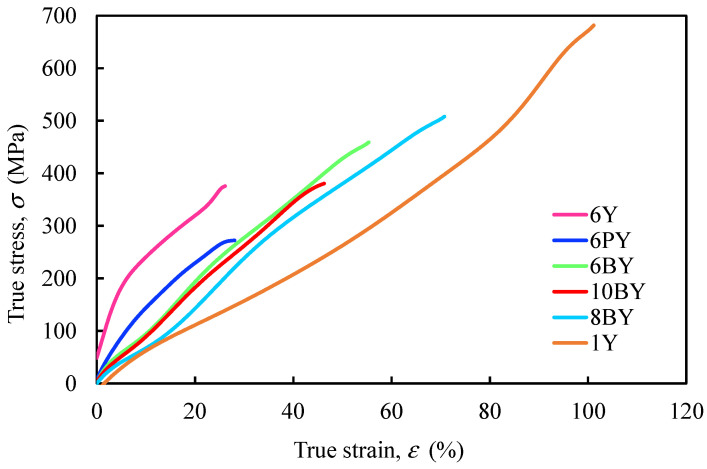
Average true stress–strain curves of the different PVA fibrous architectures.

Average true stress–strain curves were also obtained (Figure 11) from 28 results (Figure 8) to emphasise the existing differences of each fibrous architecture and determine the parameters for the mechanical characterisation of the material (Table 2), such as the Young modulus (*E*), the tensile strength (*σ*^u^), and the ultimate strain (*ε*^u^), which are both true values. 

### 3.6. Creep Tensile Tests

**Figure 12:**
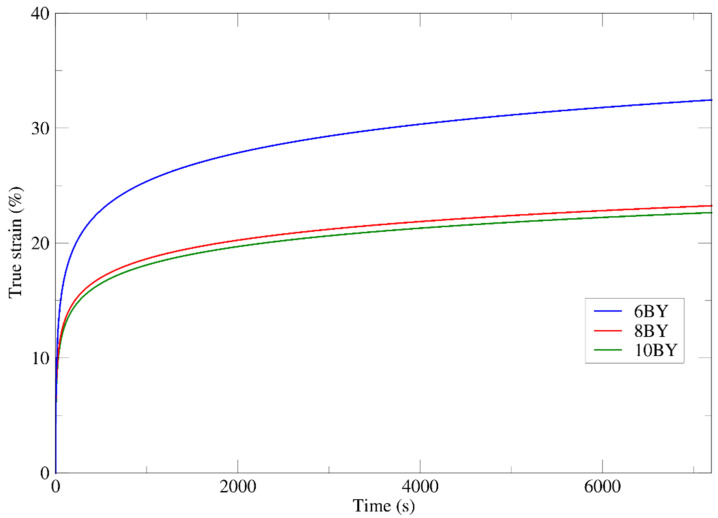
Mean curves of tensile creep tests of PVA 6BY, 8BY, and 10BY.

Creep is an important test to describe the viscoelastic performance of materials. It can be defined as the increase in the strain under constant applied stress (*σ*_0_) over a period (i.e., time of creep). To carry out those tests, input *σ*_0_ stress values of 63.76 MPa, 67.12 MPa, and 56.45 MPa (i.e., 16% of the corresponding tensile strengths) were imposed to each braided architecture 6BY, 8BY, and 10BY, respectively. The analysis of the attained mean curves for true tensile strain vs. time in Figure 12 (from 10 specimens per architecture) allows us to conclude that the 6BY structure shows a significant evolution of the tensile strain (within 7200 s), stabilising at 32.5%. The 8BY and 10BY structures show similar behaviour, tending to approximately 24% of true strain. Taking into account the difference between the value of *ε*^u^ (Table 2) and the strain (*ε*) obtained in the creep test (time of creep), it can be concluded that this difference is smaller in the 6BY configuration (13.7%) and larger in 8BY (29.4%). This means that the braided architecture that allows a smaller margin for further deformation until final rupture is 6BY, while the highest margin is attained for the 8BY configuration. Taking this argument into account, it turns out that the behaviour of 10BY, although identical to the 8BY architecture in the tensile creep test (Figure 12), is indeed much closer to the 6BY architecture in terms of attainable margin until final rupture (i.e., a difference of 18.1%). 

Considering the mean curves plotted in Figure 12 (from 10 valid results), three functions expressing the evolution of the true strain with the time of creep within 7200 s were extracted for each PVA braided architecture (see Table 3). 

### 3.7. Relaxation Tensile Tests

**Figure 13:**
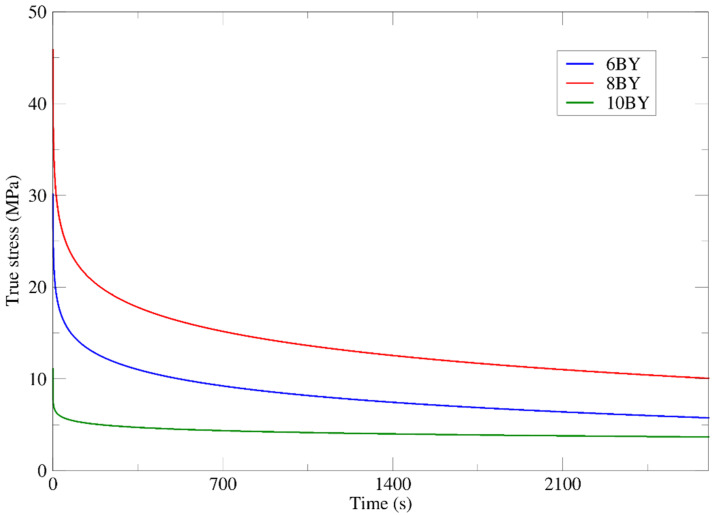
Mean curves of tensile relaxation tests of PVA 6BY, 8BY, and 10BY.

Relaxation can be defined as the decrease in the stress under constant applied strain (*ε*_0_) over a period of time. The applied strain has been set to 5%, 8.5%, and 8%, respectively, for 6BY, 8BY, and 10BY architectures (Table 4). These values were considered bearing in mind the strain value corresponding to 16% of the tensile strength (*σ*^u^) for each braided structure. Figure 13 shows the relaxation responses (mean curves) that have been obtained for the 6BY, 8BY, and 10BY fibrous architectures over 2700 s. A significant evolution of the tensile stress over the test time for 6BY is observed from approximately 30 to 5 MPa. Although a significant strength loss is visible in this test, it turns clear that a residual strength is attained (approximately 5 MPa). The analysis of the 8BY structure shows similar behaviour in the tensile stress over time. The corresponding decrease in tensile stress occurred between 45 MPa and approximately 10 MPa. Regarding the 10BY structure, the decrease in stress is considerably smaller than the preceding ones (i.e., 11.25 to 3.75 MPa), revealing a higher capacity to withstand the applied stress over time. Comparing the results of the relaxation tests for each braid architecture, it can be concluded that the 10BY configuration is the one that presents the least strength loss (from 11 to 3 MPa). In turn, the highest strength loss has been revealed by 8BY (45 to 10 MPa) over time.

### 3.8. DMA Analyses

**Figure 14:**
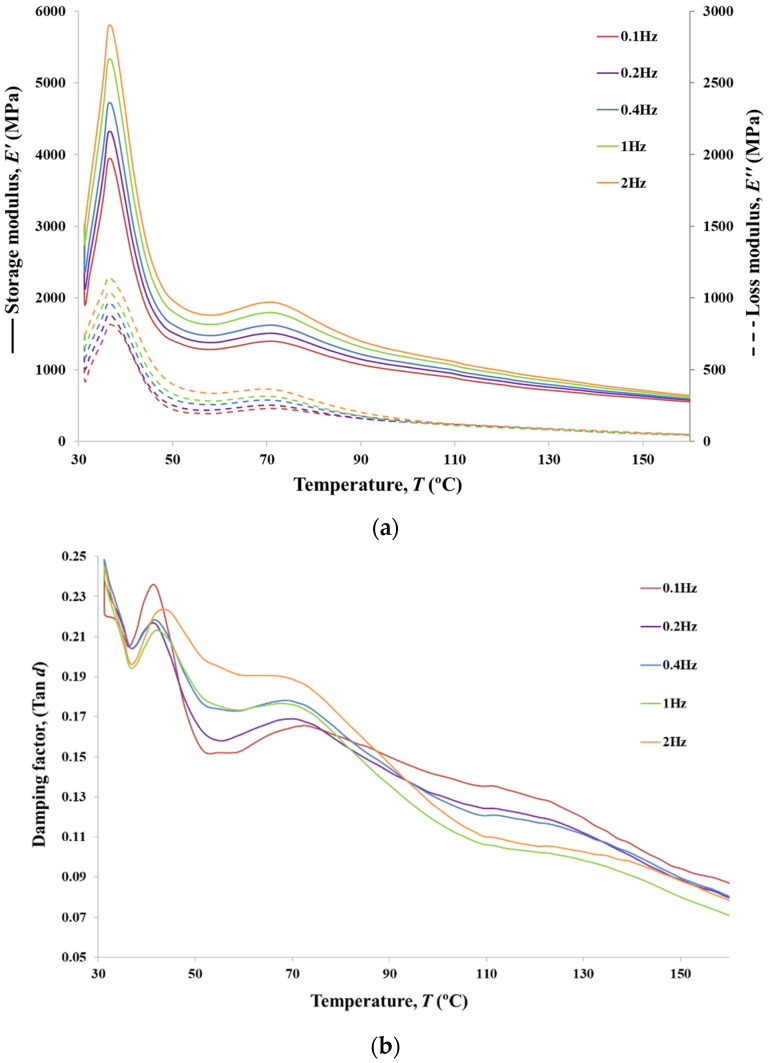
Storage and loss moduli (**a**), and tangent loss (**b**) for PVA 6BY.

**Figure 15:**
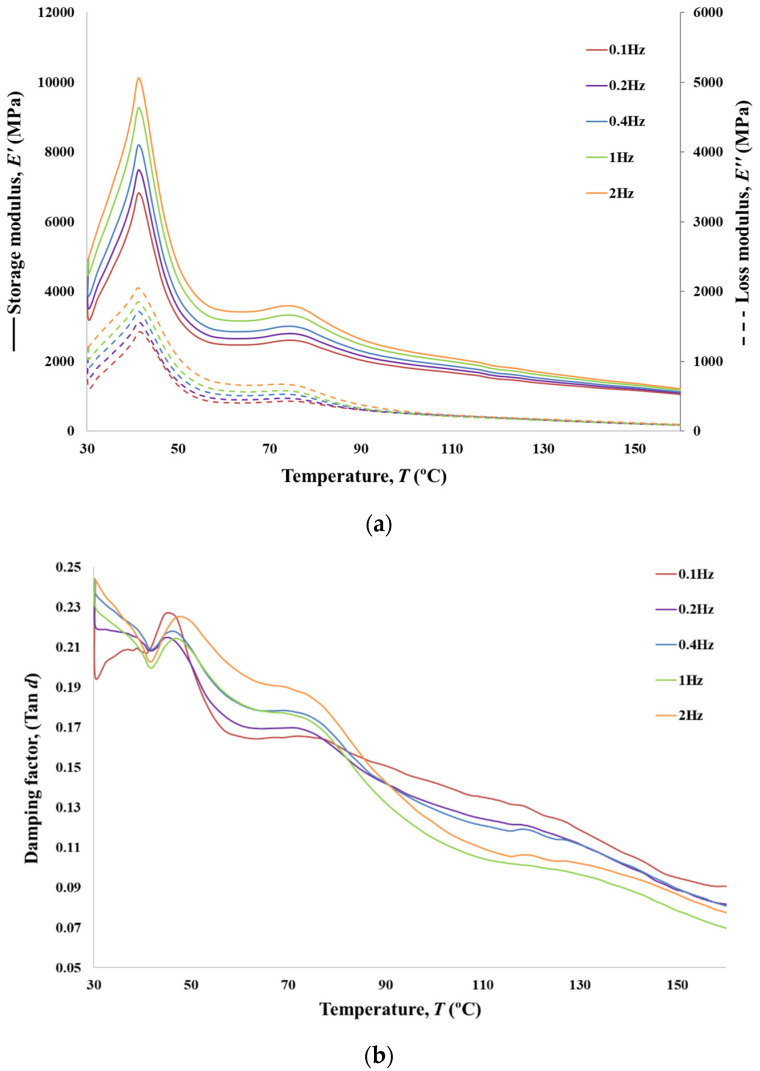
Storage and loss moduli (**a**), and tangent loss (**b**) for PVA 8BY.

**Figure 16:**
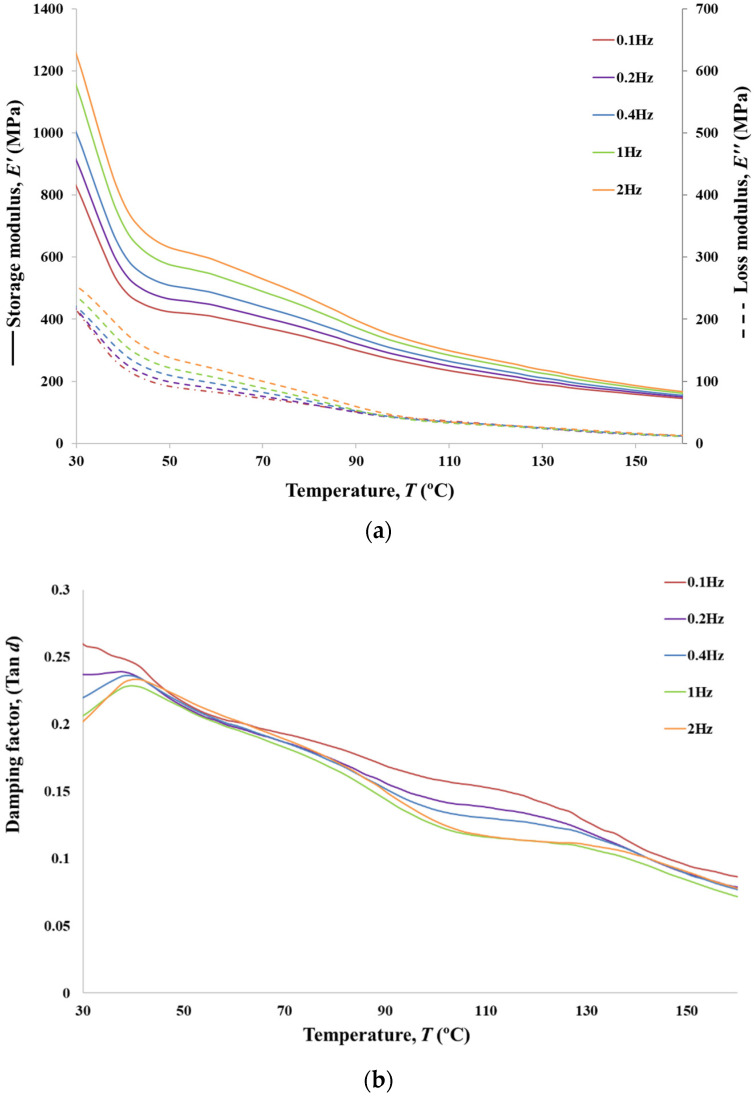
Storage (**a**) and loss moduli, and tangent loss (**b**) for PVA 10BY.

In addition to the viscoelastic characterisation (creep and relaxation), DMA tests were performed for the analysed PVA braided yarns. The presented values for tensile modulus were collected over a range of frequencies from 0.1 to 2 Hz in synthetic oscillation mode for 6BY (Figure 14), 8BY (Figure 15), and 10BY (Figure 16). Temperature dependence of the loss tangent (i.e., tan*δ*), storage, and loss moduli (*E*′ and *E*″, respectively) were measured in the temperature range of 30–160 °C at a heating rate of 3 °C min^−1^. Both *E*′ and *E*″ curves present similar transition peaks for all the tested braided yarns (Figure 14, Figure 15 and Figure 16), except for the 10BY system. The 6BY and 8BY curves show a very similar pattern that can be divided into three different regions associated with molecular motion and subsequent changes in the moduli values. The first one is a clear peak at 37 °C and 42 °C for the 6BY and 8BY systems, respectively, in both *E*′ and *E*″. The second one is a broader and lower peak at 71 °C and 75 °C for the 6BY and 8BY, respectively, in both the *E*′ and *E*″. The last region is characterised by a constant decrease in the moduli with a diminishing frequency difference. Nonetheless, the 10BY system showed a similar trend, the second peak is barely visible, and the first one appears at a lower temperature in both moduli. These results show that the higher the loading frequency, the higher the *E*′ and *E*″. It should be noted that *E*′ is always four to six times higher than *E*″, but the differences are in the same order of magnitude. 

The unusual growth in the *E*′ between 30 and 37 °C can be attributed to a cold crystallisation effect of the PVA Mintval^®^ fibres obtained by the melt spinning process of Exceval^®^ polymer from Kuraray. A cold crystallisation peak is not a surprise, since spun fibres have a lower degree of crystallisation than drawn or staples fibres. Consequently, the modulus of a semicrystalline polymer will result in a higher value than the analogous amorphous polymer [38]. A cold crystallisation effect, in this case, creates a semicrystalline PVA fibre consisting of amorphous and crystalline regions. The value of *E*′ rapidly decreases, due to melting of crystal form, after reaching a peak that is concomitant with a negative peak in tan*δ*. This indicates a clear elastic and solid-like behaviour [38]. The value of tan*δ* at these physiological temperatures varies from 0.19 to 0.25, which confirms that the system is basically elastic under tensile load, presenting a small, though not negligible, viscosity. After this peak, a dramatic decrease *E*′ and *E*″ is observed, corresponding to peaks at 41 °C and 47 °C in tan*δ* of the 6BY and 8BY, respectively. These peaks could be associated with the beta relaxation *T*_β_, which is related to the motion of backbone segments of PVA side chains where acetate groups or crosslinking species are present, or to the glass transition temperature *T*_g_ of the non-hydrolysed poly(vinyl acetate). 

Since PVA is usually obtained by the hydrolysis of poly(vinyl acetate) [39,40], these results suggest that the PVA used in these fibres is not fully hydrolysed. The movement of the side chains of PVA can also be improved by the temperature increase or moisture existence, resulting in an increase in free volume, easing the side chains’ movement [24]. This transition showed a tan*δ* peak shift to higher temperature only at the highest frequency of 2 Hz, suggesting that the peak can be associated with the *T*_g_ of poly(vinyl acetate). However, that selected frequency is too low to show an explicit Arrhenius behaviour as a function of oscillation frequency, not allowing the determination of the activation energy [41]. The second lower and the broader peak is associated with the *T*_g_ of PVA, which is an alpha transition (*T*_α_) that represents the primary motion of the PVA main chain in the amorphous areas in the polymer matrix [24]. In the 10BY fibrous structure, the PVA *T*_g_ is barely visible, and the *T*_β_ appears at a lower temperature in both moduli. The fibre structural effect can explain this behaviour. The reduction of peak amplitude and the increase in peak within tan*δ* could be associated with a decrease in polymer chain mobility by restricting both side-chains rotations and main backbone movements, which could also be attributed to the more rigid 10BY architecture. 

DMA, in this case, is not only able to provide information about the behaviour of the amorphous and crystalline regions but also quantify the degree of the PVA chain confinement due to the fibre architecture and filament numbers observing the reduction of the tan*δ* peak amplitude. The higher rigidity of the system is also proved by the fact that no shift in temperature peak in tan*δ* at the highest frequency of 2 Hz is detected. 

The region between 90 and 130 °C in tan*δ* is characterised by a frequency-dependent with a more elastic response (lower tan*δ*) at high frequencies. Since the rate of deformation and the sample response is strongly related to the relaxation phenomenon, this indicates the clear viscoelastic nature of PVA fibre. At higher temperatures, up to 160 °C, tan*δ* continues to decrease. However, the response tends to converge to a single frequencies-independent value, which indicates that the polymer is becoming more molecular-oriented even if its elasticity continues to increase.

## 4. Conclusions

The performed extensive analysis of polyvinyl alcohol (PVA) is a fundamental task to understand the potentialities of this material in applications requiring the employment of PVA yarns (Y) and braided yarns (BY). 

PVA architectures were highlighted by scanning electron microscopy (SEM), rendering it possible to show the morphology and surface texture of those structures. This technique allowed verification that the transversal section for one PVA yarn (1Y) is constituted by six filaments. 

A thermogravimetric analysis (TGA) was applied to 1Y PVA with a range of temperatures from 25 to 500 °C to investigate the corresponding mass decomposition. The results showed that the degradation of this polymer suffers from 80% of mass loss at 385 °C peak corresponding to the primary chain dehydration reaction, after which tiny residues of 5% remain at the end of the analysis (500 °C). 

Differential thermal analysis (DTA) was executed to measure the amount of heat required to increase the temperature of the 1Y PVA sample. The glass transition temperature (*T*_g_) of PVA was reached at 76 °C. The corresponding crystalline melting temperature (*T*_m_) was observed at 210 °C. The crosslinking degradation was observed at two expressive peaks at 330 and 370 °C. 

Fourier-transform infrared spectroscopy (FTIR) analysis was performed, confirming the identity of the chemical compounds present in the PVA 1Y structure. 

Monotonic tensile tests were executed to evaluate quasi-static properties of the PVA structures with a different number of fibres (6 to 360), showing higher tensile strength (*σ*^u^) for the PVA braided yarns structures. Monotonic tests proved that PVA braided yarns are more viable than non-braided, since an enhancement of their mechanical properties has been attained. 

The viscoelastic characterisation was executed following creep and relaxation experimental protocols for the three types of braided structures (6, 8, and 10 yarns). According to creep tests, the 6BY PVA structure presents the most deformable behaviour, while the 8BY PVA structure is the least deformable. On the order hand, the results from relaxation tests showed that the 8BY PVA structure presents the most strength loss and the 10BY PVA architecture verified the minimum loss strength. 

Dynamic mechanical analysis (DMA) characterisation was executed to determine the viscoelastic properties of the PVA braided yarns. The graphic representing the evolution of the storage and loss moduli (*E*′ and *E*″, respectively) revealed similar transition peaks for all braided yarns, except for the 10BY system. In addition, the *E*′ value is always four to six times higher than *E*″ in the same order of magnitude.

Among the possible applications of such fibrous systems are bone fracture immobilisation devices, which could be customised with a proper fibrous architecture of PVA according to bone fracture configuration, fragment size, and location. Those solutions would benefit from intrinsic PVA features such as biocompatibility, biodegradability, and odourlessness, while preserving the required mechanical performance of the fastening system to allow the regeneration of bone tissue throughout the clinical recovery period. This would give the opportunity for the development of less-intrusive bone fixation systems to offer an adequate response to temporary injuries, dispensing further revision processes.

## Figures and Tables

**Table 1:** Results obtained in tensile tests with the adopted PVA architectures. Values in parenthesis represent the coefficient of variation in percentage.

Structure	No. of Filaments/Fibres	Braid Angle (°)	Stiffness, *K*_0_ (N/mm)	Ultimate Load, *P*_u_ (N)	Displacement at Rupture, *δ*_u_ (mm)
1Y	6	-	0.01 (14%)	0.64 (8%)	129.24 (17%)
6PY	36	-	0.21 (31%)	3.64 (8%)	46.95 (13%)
6Y	36	-	0.57 (21%)	4.52 (20%)	20.90 (26%)
6BY	216	18.198	1.35 (23%)	27.05 (7%)	59.12 (17%)
8BY	288	22.086	0.99 (27%)	35.26 (7%)	71.35 (23%)
10BY	360	25.085	1.82 (23%)	41.62 (11%)	52.65 (17%)

**Table 2:** Mean values obtained in tensile loading for analysed PVA structures. Values in parenthesis represent the coefficient of variation in percentage.

Structure	No. of Filaments/ Fibres	Area, *A* (mm^2^)	Young Modulus, *E* (MPa)	Strength, *σ*^u^ (MPa)	Ultimate Strain, *ε*^u^ (%)
1Y	6	0.003	6.029 (25%)	486.872 (27%)	82.515 (23%)
6PY	36	0.018	14.266 (27%)	296.686 (11%)	38.396 (12%)
6Y	36	0.018	33.934 (11%)	316.180 (8%)	19.569 (16%)
6BY	216	0.108	14.217 (22%)	398.501 (12%)	46.235 (14%)
8BY	288	0.144	7.535 (30%)	419.473 (14%)	53.407 (18%)
10BY	360	0.181	10.894 (21%)	352.788 (16%)	42.072 (15%)

**Table 3:** Mean true strain within 7200 s for PVA braided architecture (*σ*_0_ = 0.16 *σ*^u^).

Structure	Applied Stress (MPa)	Strain (%)
6BY	63.76	*ε* = 0.5578875 + 3.5897556 ln(*t*)
8BY	67.12	*ε* = 2.4604387 + 2.3397383 ln(*t*)
10BY	56.45	*ε* = 2.1538739 + 2.307223 ln(*t*)

**Table 4:** Mean true stress within 2700 s for PVA braided architectures (*ε*_0_ = *ε* (0.16 *σ*^u^).

**Structure**	**Applied Strain (%)**	**Stress (MPa)**
6BY	5	*σ* = 26.31123349 − 2.5757711 ln(*t*)
8BY	8.5	*σ* = 40.012435 − 3.7909087 ln(*t*)
10BY	8	*σ* = 7.7098721 − 0.51067574 ln(*t*)

## Data Availability

The raw/processed data required to reproduce these findings can be shared at this time, as the data also are part of an ongoing study.
